# Reproducibility of cine displacement encoding with stimulated echoes (DENSE) cardiovascular magnetic resonance for measuring left ventricular strains, torsion, and synchrony in mice

**DOI:** 10.1186/1532-429X-15-71

**Published:** 2013-08-27

**Authors:** Christopher M Haggerty, Sage P Kramer, Cassi M Binkley, David K Powell, Andrea C Mattingly, Richard Charnigo, Frederick H Epstein, Brandon K Fornwalt

**Affiliations:** 1 Departments of Pediatrics, Physiology and Medicine, University of Kentucky, Lexington, KY, USA; 2 Department of Biomedical Engineering, University of Kentucky, Lexington, KY, USA; 3 Department of Biostatistics, University of Kentucky, Lexington, KY, USA; 4 Departments of Biomedical Engineering and Radiology, University of Virginia, Charlottesville, VA, USA

**Keywords:** Cardiovascular magnetic resonance, DENSE, Strain, Reproducibility, Mice, Heart

## Abstract

**Background:**

Advanced measures of cardiac function are increasingly important to clinical assessment due to their superior diagnostic and predictive capabilities. Cine DENSE cardiovascular magnetic resonance (CMR) is ideal for quantifying advanced measures of cardiac function based on its high spatial resolution and streamlined post-processing. While many studies have utilized cine DENSE in both humans and small-animal models, the inter-test and inter-observer reproducibility for quantification of advanced cardiac function in mice has not been evaluated. This represents a critical knowledge gap for both understanding the capabilities of this technique and for the design of future experiments. We hypothesized that cine DENSE CMR would show excellent inter-test and inter-observer reproducibility for advanced measures of left ventricular (LV) function in mice.

**Methods:**

Five normal mice (C57BL/6) and four mice with depressed cardiac function (diet-induced obesity) were imaged twice, two days apart, on a 7T ClinScan MR system. Images were acquired with 15–20 frames per cardiac cycle in three short-axis (basal, mid, apical) and two long-axis orientations (4-chamber and 2-chamber). LV strain, twist, torsion, and measures of synchrony were quantified. Images from both days were analyzed by one observer to quantify inter-test reproducibility, while inter-observer reproducibility was assessed by a second observer’s analysis of day-1 images. The coefficient of variation (CoV) was used to quantify reproducibility.

**Results:**

LV strains and torsion were highly reproducible on both inter-observer and inter-test bases with CoVs ≤ 15%, and inter-observer reproducibility was generally better than inter-test reproducibility. However, end-systolic twist angles showed much higher variance, likely due to the sensitivity of slice location within the sharp longitudinal gradient in twist angle. Measures of synchrony including the circumferential (CURE) and radial (RURE) uniformity of strain indices, showed excellent reproducibility with CoVs of 1% and 3%, respectively. Finally, peak measures (e.g., strains) were generally more reproducible than the corresponding rates of change (e.g., strain rate).

**Conclusions:**

Cine DENSE CMR is a highly reproducible technique for quantification of advanced measures of left ventricular cardiac function in mice including strains, torsion and measures of synchrony. However, myocardial twist angles are not reproducible and future studies should instead report torsion.

## Background

The ability to make advanced measurements of myocardial function (e.g., strains and strain rates) from cardiac imaging is a potentially valuable tool in clinical assessment of ventricular function. These indices have been shown to correlate strongly with left ventricular systolic function and contractility [1-3] and predict cardiac mortality [3-5]. Furthermore, abnormalities in such advanced measures of cardiac function may precede global myocardial dysfunction [6], which suggests they may provide a useful early marker for diagnosing and treating disease.

Measuring strains with cardiovascular magnetic resonance (CMR) has been accomplished through numerous techniques including tissue tagging [7,8], phase contrast velocity mapping [9,10], and displacement encoding with stimulated echoes (DENSE) [11,12]. DENSE in particular has the distinct advantage of encoding tissue displacements into the signal phase, and thus provides for straightforward strain calculation with high spatial resolution. Therefore, DENSE has been used to assess myocardial function and mechanics in both humans [13,14] and mice [15,16].

A critical benchmark for the success and usefulness of any measurement technique is its reproducibility; that is, how much variation is observed between values measured both at different time points (inter-test) and by different observers (inter-observer). The inter-test and inter-observer reproducibility for quantification of advanced cardiac function in mice has not been evaluated, which represents an important knowledge gap to address for ensuring the future clinical and translational research utility of the method. We hypothesized that cine DENSE CMR would show excellent inter-test and inter-observer reproducibility for advanced measures of left ventricular (LV) function in mice. We tested this hypothesis in the context of mouse models with both normal and abnormal (obesity-induced dysfunction) ventricular function.

## Methods

### Mouse models

Nine 12-week-old C57BL/6 mice were randomized to either a high fat diet *ad libitum*, with 60% of calories from fat (Research Diets #D12492) or a low fat diet with 10% of calories from fat (Research Diets #D12450B). Animals were housed in ventilated cages in a temperature-controlled room with a 14:10 light:dark cycle and provided with enrichment in the form of acrylic huts and nesting material. All animal procedures conformed to Public Health Service policies for humane care and use of animals, and all procedures were approved by the institutional animal care and use committee at the University of Kentucky. Notably, differences in myocardial strains, torsion, and synchrony between these groups have previously been reported [17].

### Animal preparation

Imaging was performed 5 months after starting the diet. Animals were anesthetized with isoflurane using a precision vaporizer delivering 1.5-2.5% isoflurane in oxygen at a rate of 1.0 L/min. Once anesthetized, three legs were shaved for placement of cutaneous ECG electrodes required for cardiac gating. A diaphragm to sense breathing was placed under the abdomen for respiratory gating in order to minimize motion artifact. A rectal thermometer was placed to monitor core temperature. During scanning, all vital signs including heart rate, respiratory rate and core temperature were continuously monitored with a fiber optic system (SA Instruments, Inc, Stony Brook, NY). Body temperature was maintained between 36 and 37 degrees Celsius with a heated water blanket.

### CMR

**Figure 1:**
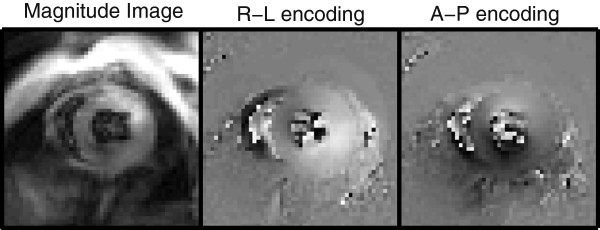
**Representative output from mid-ventricular short axis slice using the DENSE acquisition protocol.** Three images are created: a magnitude image (left), a phase image encoded in the ‘x’ or right-left (R-L) direction (middle) and a second phase image encoded in the ‘y’ or anterior-posterior (A-P) direction (right).

CMR was performed on a 7-Tesla BrukerClinScan system (Bruker, Ettlingen, Germany) equipped with a 4-element phased array cardiac coil and a gradient system with a maximum strength of 450 mT/m and a maximum slew rate of 4500 mT/m/s. Image acquisition has been described in detail previously [11,18]. Briefly, the CMR tissue tracking method known as cine Displacement Encoding with Stimulated Echoes (DENSE) was utilized. Immediately after an electrocardiogram R-wave trigger detection, which marks the depolarization of the ventricles and onset of contraction, a displacement encoding module consisting of radiofrequency and gradient pulses was applied, which stores position-encoded longitudinal magnetization. This initial encoding was followed by successive applications of a readout module, consisting of a radiofrequency excitation pulse, a displacement un-encoding gradient, and an interleaved spiral k-space trajectory. This sequence creates 3 images: a magnitude image and two phase images independently encoded for ‘x’ and ‘y’ displacements, respectively (Figure 1). A total of 15–20 frames per cardiac cycle using both cardiac and respiratory gating were acquired with a repetition time of 7.1 ms. Other relevant acquisition parameters included: field of view = 32 mm, matrix = 128 × 128, slice thickness = 1 mm, echo time = 0.67 ms, number of averages = 3, number of spiral interleaves = 36, and displacement encoding frequency = 0.8 cycles/mm. Each two-dimensional image acquisition took approximately 6–9 minutes depending on the heart rate (usually 400–600 beats per minute) and respiratory rate (usually 90–140 breaths per minute) of the animal.

We acquired 3 short-axis images and 2 long-axis images for each mouse. The long-axis images consisted of a standard apical 4-chamber view and a 2-chamber view perpendicular to the 4-chamber view. The short-axis images were planned perpendicular to the 4-chamber long-axis image. Specifically, the apical and basal slice positions were placed at a distance 20% of the end-systolic ventricular length above and below the mid-ventricle, which itself was defined as 50% of the measured end-systolic length.

### Image analysis

**Figure 2:**
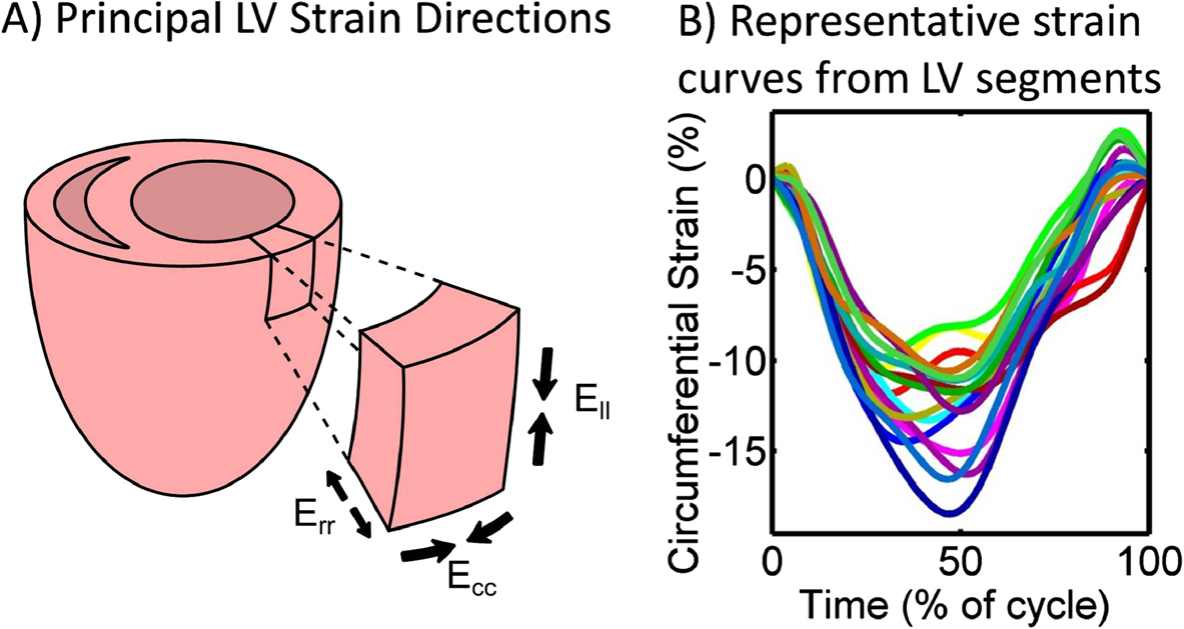
**Segmental analysis of strains in three orthogonal directions of the LV. A)** Definition of strain components for a given section of LV myocardium: radial (rr), circumferential (cc), and longitudinal (ll). Arrows denote bulk myocardial strain directions during LV contraction. **B)** Representative circumferential strain vs. time curve over the cardiac cycle. Each curve represents one component of the standard 16 segment LV model. Negative values of strain denote shortening with respect to the end diastolic baseline.

The displacement-encoded phase images were used to derive advanced quantitative measures of cardiac function offline using custom software written in MATLAB (Mathworks, Inc., Natick, MA). The basic steps included semi-automated motion-guided segmentation of the myocardium from the blood pool and surrounding tissue, phase unwrapping, and tissue tracking to derive the actual displacement of each pixel throughout the cardiac cycle [19,20]. User correction of the automated segmentation could be performed as needed. The displacement vectors were then decomposed into orthogonal directions with respect to the LV, as shown in Figure 2A: radial, circumferential and longitudinal. Global cardiac circumferential and radial strain curves were derived from averaging the strain curves of each of the 16 standardized segments of the left ventricle [21] (see Figure 2B for example of circumferential strain curves for the 16 segments). In addition, the myocardium was automatically divided into thirds (‘subendocardium’, ‘mid-wall’, and ‘subepicardium’) for individual evaluation of the transmural layers. For long-axis data, the apical segments were excluded from analysis.

The strains were used to quantify left ventricular synchrony using the circumferential and radial uniformity ratio estimate indices (CURE and RURE) [22]. Twist was defined by the angle between radial lines connecting the LV centroid on a given slice to the voxel of interest between the time point of interest (e.g., end systole) and end diastole [16]. A positive angle denotes counter-clockwise rotation viewed from the standard imaging perspective (apex/foot). Torsion was defined as the difference in twist angle between the basal and apical slices normalized by the long-axis epicardial length of the left ventricle at end-diastole (average of lengths measured on the 2-chamber and 4-chamber images) [23].

### Reproducibility assessment

Two different comparisons were sought: 1) Inter-test reproducibility (i.e., the change in a given quantity through separate, independent measurements on the same animal) and 2) inter-observer reproducibility (i.e., the change in a given quantity through independent analyses of a single measurement by multiple observers). For the inter-test assessment, each of the 9 mice was imaged with the same protocol on two different days spaced two days apart and the post-processing was carried out by a single user (#1). For the inter-observer test, a second investigator (#2) repeated the post-processing analyses for all 9 mice on the data set from the first day. For all comparisons, reproducibility was assessed using a modified mean coefficient of variation (CoV), which compares the measurement variability of the given variable, X, over the N mice to its absolute magnitude, as follows:

$$\mathrm{CoV}=\frac{\sum_{i=1}^{N}\left[\mathrm{St}.\mathrm{Dev}.{\left(X_{\mathrm{Obs}.1\;}X_{\mathrm{Obs}.2}\right)}_{i}\right]}{\left|\sum_{i=1}^{N}\left[{\left(\left(X_{\mathrm{Obs}.1}+X_{\mathrm{Obs}.2}\right)/2\right)}_{i}\right]\right|}$$

CoV results less than or equal to 20% were considered reproducible.

## Results

### Strain and strain rate

The strain and strain rate results are summarized in Tables 1 and 2, respectively. The inter-observer reproducibility can be assessed by comparing the first two columns, while the inter-test reproducibility data are in columns two and three. Strains and strain rates in the radial, circumferential and longitudinal directions are reported with each direction decomposed into endocardial, mid-myocardial, and epicardial segments in addition to the ‘global’ average value. Additionally, the strain rates are separated into systolic and diastolic components. The radial direction had both the largest magnitudes and standard deviations in strains and strain rates, while the longitudinal and circumferential values were similar in magnitude. Heterogeneity in response across the myocardial layers was also seen.

**Table 1:** Summary (mean ± standard deviation) of peak myocardial strain results in the radial, circumferential, and longitudinal directions

		**Observer 2, Day 1**	**Observer 1, Day 1**	**Observer 1, Day 2**
**Peak radial strain (%)**	Endo	32 ± 6	35 ± 5	31 ± 6
	Mid	36 ± 7	34 ± 5	33 ± 6
	Epi	23 ± 10	21 ± 7	20 ± 4
	Global	32 ± 6	31 ± 4	29 ± 4
**Peak circumferential strain (%)**	Endo	−16 ± 2	−16 ± 1	−15 ± 1
	Mid	−11 ± 2	−12 ± 2	−11 ± 1
	Epi	−8 ± 2	−8 ± 2	−8 ± 1
	Global	−12 ± 2	−12 ± 2	−11 ± 1
**Peak longitudinal strain (%)**	Endo	−12 ± 1	−12 ± 1	−12 ± 1
	Mid	−11 ± 1	−11 ± 2	−11 ± 1
	Epi	−10 ± 1	−10 ± 1	−10 ± 1
	Global	−11 ± 1	−11 ± 1	−11 ± 1

**Table 2:** Summary (mean ± standard deviation) of peak myocardial strain rate results

**Systolic**		**Observer 2, Day 1**	**Observer 1, Day 1**	**Observer 1, Day 2**
**Peak radial strain rate (%/ms)**	Endo	12 ± 2	12 ± 3	13 ± 3
	Mid	12 ± 3	12 ± 3	12 ± 2
	Epi	9 ± 2	9 ± 3	9 ± 2
	Global	11 ± 2	10 ± 2	11 ± 2
**Peak circumferential strain rate (%/ms)**	Endo	−6 ± 1	−7 ± 1	−7 ± 1
	Mid	−5 ± < 1	−5 ± 1	−5 ± < 1
	Epi	−3 ± < 1	−3 ± < 1	−3 ± < 1
	Global	−5 ± < 1	−5 ± < 1	−5 ± < 1
**Peak longitudinal strain rate (%/ms)**	Endo	−6 ± 2	−5 ± 1	−5 ± 2
	Mid	−5 ± 1	−5 ± 1	−5 ± 1
	Epi	−4 ± 1	−4 ± 1	−5 ± 1
	Global	−5 ± 1	−5 ± 1	−5 ± 1
**Diastolic**		**Observer 2, Day 1**	**Observer 1, Day 1**	**Observer 1, Day 2**
**Peak radial strain rate (%/ms)**	Endo	−12 ± 3	−13 ± 4	−12 ± 4
	Mid	−12 ± 4	−12 ± 3	−12 ± 5
	Epi	−9 ± 3	−8 ± 3	−8 ± 2
	Global	−11 ± 3	−11 ± 3	−10 ± 3
**Peak circumferential strain rate (%/ms)**	Endo	6 ± 2	6 ± 1	6 ± 1
	Mid	4 ± 1	4 ± 1	4 ± 1
	Epi	3 ± < 1	3 ± 1	3 ± 1
	Global	4 ± 1	4 ± 1	4 ± 1
**Peak longitudinal strain rate (%/ms)**	Endo	5 ± 2	4 ± 1	5 ± 2
	Mid	4 ± 1	4 ± 1	4 ± 2
	Epi	4 ± 1	4 ± 1	4 ± 2
	Global	4 ± 1	4 ± 1	4 ± 2

**Figure 3:**
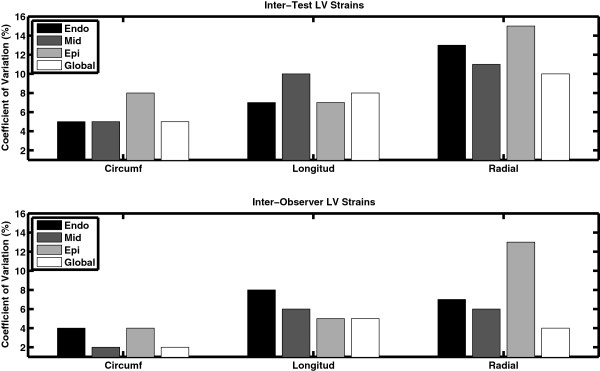
**Coefficients of variation for the inter-test (top) and inter-observer (bottom) analyses of LV strains.** The data are broken down by strain direction (either circumferential [‘Circumf’], longitudinal [‘Longitud’], or radial), and by location within the myocardium (endocardium [‘Endo’], mid-myocardium [‘Mid’], epicardium [‘Epi’], or averaged across all layers [‘Global’]). Excellent reproducibility is seen in all cases with CoVs ≤ 15%.

**Figure 4:**
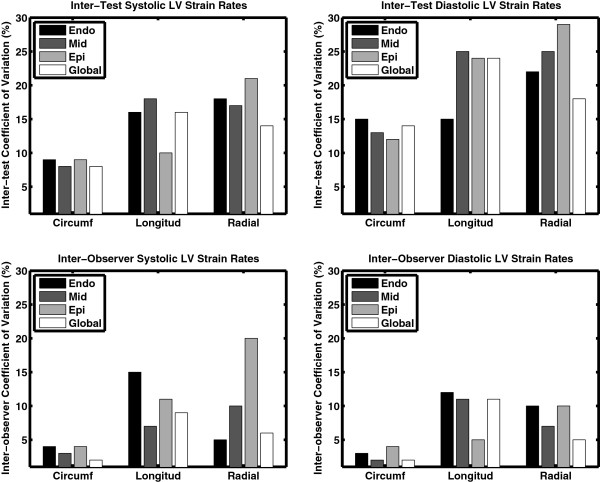
**Coefficients of variation for the inter-test (top row) and inter-observer (bottom row) analyses of LV strain rates during both systole (left column) and diastole (right column).** The data are broken down by strain direction (either circumferential [‘Circumf’], longitudinal [‘Longitud’], or radial), and bytransmural location (endocardium [‘Endo’], mid-myocardium [‘Mid’], epicardium [‘Epi’], or averaged across all layers [‘Global’]). The data, particularly for Global averages, show that these measures are reproducible (CoVs ≤ 20%), with the exception of the inter-test diastolic longitudinal strain rate.

Figure 3 presents the inter-test (top) and inter-observer (bottom) CoV for the LV strain results. In both cases, the measures were highly reproducible with all CoVs ≤ 15%. In general, the inter-observer variability was lower than inter-test, while the circumferential strain was less variable than the longitudinal or radial strains. Similarly, Figure 4 shows the CoVs for the LV strain rates. The inter-observer reproducibility was again strong, particularly the diastolic rates, with all CoVs ≤ 20%. For the inter-test comparisons, the global averages were reproducible (with the possible exception of longitudinal diastolic strain rate) but analyses of individual transmural layers were more variable, particularly in the radial and longitudinal directions (CoV > 20%).

### Twist angles and torsion

Table 3 provides a summary of the peak magnitudes and rates of myocardial twist and torsion. The twist data are reported for three short axis slices positioned at the apex, mid-ventricle, and base. As expected, the direction of the myocardial twist angle changed among these slices, going from a large positive twist at the apex, to near 0 in the mid-ventricle, and to a smaller (as compared to the apical magnitude) negative twist at the base during systole.

**Table 3:** Magnitudes and rates (mean ± standard deviation) of myocardial twist and torsion

		**Obs. 2, Day 1**	**Obs. 1, Day 1**	**Obs. 1, Day 2**	**Inter-test CoV (%)**	**Inter-Obs CoV (%)**
**End systolic twist (°)**	Basal	−1.9 ± 1.3	−2.2 ± 1.4	−2.7 ± 1.1	34	13
	Mid	0.5 ± 1.7	0.5 ± 1.8	−0.1 ± 1	547	24
	Apical	4.6 ± 2.6	4.5 ± 2.4	3.7 ± 1.3	34	6
**Peak systolic twist rate (°/ms)**	Basal	−90 ± 30	−100 ± 30	−130 ± 30	26	8
	Mid	120 ± 70	120 ± 70	130 ± 50	32	6
	Apical	180 ± 160	170 ± 60	190 ± 40	20	4
**Peak diastolic twist rate (°/ms)**	Basal	50 ± 50	60 ± 50	70 ± 80	82	17
	Mid	−100 ± 40	−100 ± 40	−70 ± 40	35	7
	Apical	−160 ± 40	−150 ± 40	−160 ± 50	30	5
**Peak torsion (°/cm)**		7.7 ± 2.0	7.9 ± 1.8	7.7 ± 1.1	12	5
**Peak systolic torsion rate (°/cm/ms)**		240 ± 40	250 ± 40	270 ± 80	22	6
**Peak diastolic torsion rate (°/cm/ms)**		−200 ± 70	−210 ± 90	−230 ± 80	25	10

**Figure 5:**
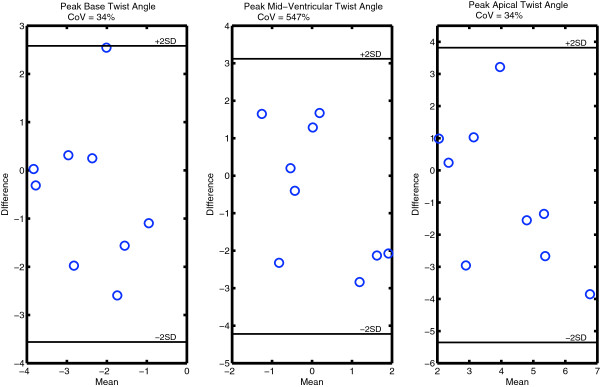
**Bland-Altman plots of the inter-test differences in end systolic twist angles at the ventricular base (left), mid-ventricle (middle), and apex (right).** In all cases, the limits of agreement (± 2 standard deviation lines) are large compared to the mean values being measured, which translated into poor CoVs (listed at the top of each plot). Because of the small twist angles at the mid-ventricular slice, this effect is particularly pronounced (CoV = 547%).

Table 3 also lists the inter-test and inter-observer CoV results for each measure showing that, while the inter-observer reproducibility of the LV twist angles and rates was borderline acceptable (≤ 24%), the inter-test results were poor, especially for the mid-ventricular slice (CoV > 500% in LV twist at end systole). Figure 5 shows the Bland-Altman plots for the end systolic twist data on each of the short axis slices. In each case, the 95% limits of agreement were large compared to the mean twist angles measured, particularly for the mid-ventricular slice, which had values close to 0°.

Peak torsion was highly reproducible: CoV = 12% and 5% for inter-test and inter-observer, respectively. However, peak torsion rates were not reproducible on an inter-test basis: CoV = 22% and 25% for systolic and diastolic results, respectively.

### Synchrony

Table 4 summarizes the results for the CURE and RURE indices of synchrony. In all cases, the reproducibility was excellent (CoV < 5%), particularly for CURE.

**Table 4:** Measures (mean ± standard deviation) of ventricular synchrony

		**Observer 2, Day 1**	**Observer 1, Day 1**	**Observer 1, Day 2**	**Inter-test CoV (%)**	**Inter-Obs CoV (%)**
**CURE**	Total	0.98 ± 0.02	0.98 ± 0.01	0.98 ± 0.02	1	1
	Systolic	0.99 ± 0.01	0.99 ± 0.01	0.98 ± 0.02	1	1
	Diastolic	0.98 ± 0.02	0.98 ± 0.01	0.98 ± 0.02	1	1
**RURE**	Total	0.92 ± 0.06	0.93 ± 0.03	0.92 ± 0.06	3	3
	Systolic	0.93 ± 0.05	0.94 ± 0.03	0.94 ± 0.05	3	2
	Diastolic	0.91 ± 0.07	0.92 ± 0.04	0.90 ± 0.08	4	4

### Reproducibility of obese vs. normal weight mice

To characterize the potential influence that mouse obesity had on the reproducibility data, Table 5 compares the CoV between mouse groups for selected function measures. No large or systematic differences in reproducibility were observed between groups.

**Table 5:** Differences in CoV (%) between obese and normal weight mice

	**Obese mice (n = 4)**	**Normal weight mice (n = 5)**
**Peak radial strain**	12	8
**Peak circumferential strain**	4	7
**Peak longitudinal strain**	9	7
**End Systolic basal twist angle**	35	32
**End Systolic apical twist angle**	43	32
**End Systolic mid-ventricular twist angle**	336	218
**Peak torsion**	8	15

## Discussion

We acquired and analyzed DENSE CMR data from normal and obese C57BL/6 mice on multiple days and using multiple observers to quantify the inter-test and inter-observer reproducibility characteristics of advanced measures of cardiac function (myocardial strains, twist angles, torsion, and synchrony). Our major findings are: 1) inter-observer reproducibility was generally good for all observed quantities; 2) inter-test reproducibility was also good for LV strains, torsion, and synchrony, but not for myocardial twist angles; 3) reproducibility of peak values was generally better than that of the corresponding peak rates (e.g., peak strain was more reproducible than the peak strain rate). Future studies should consider and utilize these findings in selecting analytical end points of cross-sectional or serial analyses using similar methods, particularly with respect to twist angles vs. torsion.

### Twist angles vs. torsion

Despite the fact that twist and torsion are both fundamentally measuring the angular myocardial displacement, we found that reproducibility for torsion (length normalized difference in twist between the apical and basal slices) was better than that of the twist alone. These results are consistent with a recent reproducibility study of CMR tagging in humans [24]. Additionally, Lorenz *et al*. previously reported the reproducibility of LV twist as measured by MR tagging, and found large (2.1 ± 1.6°) mean inter-study differences in three human volunteers (with reported measures ranging from -6° - 14°) [25]. As seen in Table 3, there is a large gradient in twist angles along the longitudinal length of the ventricle, so a small difference in slice position could yield different results, which limited the inter-test reproducibility. This observation is particularly true for the mid-ventricular slice, in the transition zone between clockwise and counter-clockwise rotation, where even small absolute differences can yield large percentage errors. By calculating this gradient rather than isolated twist angles, the variation in torsion is reduced, making that the preferred quantity to measure over twist. However, there are two critical features that are worth noting: 1) particular care was taken in this protocol in placing the short axis slices with respect to the ventricle and each other, which likely optimized the repeatability; and 2) torsion may or may not be similarly sensitive to slice positioning decisions depending on the characteristics of the twist gradient. At least one study has suggested that torsion is constant along the LV (i.e., the twist gradient is linear) [26], but further research, including standardization of torsion calculation methods, is needed to confirm these findings.

### Radial strain

Previous reports using feature tracking or tagging have demonstrated weaknesses in the reproducibility of radial strains [27,28], likely owing to limited spatial resolution and small numbers of pixels in the radial direction. Our finding that radial strains were the least reproducible compared to circumferential and longitudinal strains was therefore expected. However, the values obtained with DENSE (CoVs ≤ 15%) represent a substantial improvement in the reproducibility of radial strain. This improvement reflects the superiority of spatial resolution (e.g., more pixels in the radial dimension) and measurement accuracy of DENSE compared to feature tracking or MR tagging.

### Peak values vs. rates of change

In general, the CoVs for peak values were better than the respective measures of rates of change with respect to time. For example, comparing Figures 3 and 4 reveals that the peak strain coefficients were generally lower than the peak strain rate coefficients, particularly for the inter-test comparison. This is not to say that peak strain rates are not reproducible, but instead reflects the fact that estimating a derivative from an inherently noisy measurement will tend to amplify the noise in the signal [29,30].

### User influence on results

While the objective of this study was to characterize the reproducibility of DENSE measurements, there are important influences that the users have on the results of these measures that should be noted. First, slice positioning during data acquisition has a primary effect, as was particularly noted with regard to twist angle measures. Potential differences in slice placement and angle with respect to the long-axis may have contributed to some variance in the inter-test measures. However, inter-test results were favorable despite this potential influence, so future studies should use similarly rigorous and repeatable protocols for slice positioning and placement to avoid introducing additional uncertainty or errors.

A second source of user influence is the image segmentation, which was the primary motivation for quantifying inter-observer reproducibility. Our results indicate that DENSE is highly reproducible on an inter-observer basis as evidenced by the fact that (with the exception of mid-ventricle end systolic twist) all inter-observer CoVs were ≤ 20%. While this finding is a major strength of the study, it is important to note that the agreement was likely significantly strengthened by the use of the motion-guided segmentation algorithm used for the post-processing step [20]. In this scheme, the user manually defines initial segmentation contours on one of the phases that are propagated and appropriately moved to fit the borders of the rest of the cine images based on the measured displacement phase data. Fine adjustments can be made to the automated contours, but minimal changes are generally required. In the validation of this procedure presented by Spottiswoode *et al*. it was found that this motion-guided procedure had the potential to reduce segmentation errors below the limits of inter-observer reproducibility [20]. So while the present results were not completely devoid of observer-related differences, repeating the analysis with completely manual segmentation would likely have made the results less reproducible.

### Use of mouse obesity model

Although a direct comparison of normal function and obesity-mediated ventricular dysfunction was not a focus of this study, the inclusion of the obese mice in the study design was notable and important for several reasons. Even though Table 5 indicates that there were not significant differences in reproducibility of function measures between groups, the inclusion of two different function groups in the study was important to avoid a homogenous and thus easily reproducible data set. Furthermore, reproducing ‘disease’ function in mice is typically of greater importance and interest than ‘normal’ function. In that sense, obese mice offered a good disease model for the present work since they are known to have 1) cardiac remodeling and hypertrophy [31], 2) dysfunction in many of the metrics we measured [17] and 3) are theoretically more difficult to image due to problems with fat artifacts and generally poorer health/tolerance of anesthesia.

### Study limitations

We have demonstrated reproducibility of DENSE using a mouse model of LV function, and cannot directly extrapolate these results to comment on the reproducibility of DENSE in humans. Given the potential value of DENSE in clinical ventricular assessment, repeating this assessment in humans therefore represents a worthwhile exercise. However, mouse models of ventricular function are extensively used for basic science and translational studies into cardiac disease, so demonstrating the reproducibility of DENSE in this context is an important contribution.

We did not utilize a fat suppression technique during image acquisition in this study. Future studies in obese mice may benefit from fat suppression to improve image quality. However, the overall quality of the images was extremely good in both the obese and normal mice (Figure 1).

We did not directly quantify the intra-observer reproducibility from a single dataset. Doing so could provide additional insights into the influence of the motion-guided segmentation on the observed agreement between observers. However, since inter-test reproducibility was found to be uniformly lower than inter-observer, and intra-observer reproducibility would be, at worst, no less than inter-observer, it is clear that the inter-test reproducibility is the limiting factor in the overall reproducibility of DENSE.

## Conclusions

Cine DENSE CMR is a highly reproducible technique for the quantification of advanced measures of left ventricular function, including strains, torsion, and synchrony, in mice. Radial strain was generally less reproducible than circumferential strain, but the reproducibility of radial strain with DENSE was far superior to previous results reported using other techniques. Quantification of peak values was more reproducible than the associated rates of change. Myocardial twist angles are not reproducible, likely because of the high sensitivity to slice placement within the large longitudinal twist gradient that exists within the left ventricle. However, we found that torsion is reproducible, so future studies measuring myocardial twist should instead quantify torsion. These findings provide strong support for the use of these DENSE-derived measures of advanced cardiac function to identify early surrogates for dysfunction and disease and to further our understanding of mouse models of disease in translational research.

## Abbreviations

CMR: Cardiovascular magnetic resonance; DENSE: Displacement encoding with stimulated echoes; LV: Left ventricle; CURE: Circumferential uniformity ratio estimate; RURE: Radial uniformity ratio estimate; CoV: Coefficient of variation.

## Competing interests

Dr. Epstein receives research support from Siemens. None of the other authors has competing interests.

## Authors’ contributions

CH completed the data analysis and drafted the manuscript. SK and CB assisted with data collection and performed the image segmentation. DP oversaw the CMR acquisition. AM assisted with data collection and analysis, RC contributed to the statistical study design and helped to draft the manuscript. FE developed the acquisition protocols and participated in the study design. BF conceived the study, participated in its design and coordination, and helped to draft the manuscript. All authors read and approved the final manuscript.
